# Detecting Large-Scale Brain Networks Using EEG: Impact of Electrode Density, Head Modeling and Source Localization

**DOI:** 10.3389/fninf.2018.00004

**Published:** 2018-03-02

**Authors:** Quanying Liu, Marco Ganzetti, Nicole Wenderoth, Dante Mantini

**Affiliations:** ^1^Neural Control of Movement Laboratory, Department of Health Sciences and Technology, ETH Zürich, Zurich, Switzerland; ^2^Laboratory of Movement Control and Neuroplasticity, KU Leuven, Leuven, Belgium; ^3^Department of Control and Dynamical Systems, California Institute of Technology, Pasadena, CA, United States; ^4^Department of Experimental Psychology, University of Oxford, Oxford, United Kingdom

**Keywords:** electroencephalography, high-density montage, realistic head model, resting state network, functional connectivity, neuronal communication, brain imaging

## Abstract

Resting state networks (RSNs) in the human brain were recently detected using high-density electroencephalography (hdEEG). This was done by using an advanced analysis workflow to estimate neural signals in the cortex and to assess functional connectivity (FC) between distant cortical regions. FC analyses were conducted either using temporal (tICA) or spatial independent component analysis (sICA). Notably, EEG-RSNs obtained with sICA were very similar to RSNs retrieved with sICA from functional magnetic resonance imaging data. It still remains to be clarified, however, what technological aspects of hdEEG acquisition and analysis primarily influence this correspondence. Here we examined to what extent the detection of EEG-RSN maps by sICA depends on the electrode density, the accuracy of the head model, and the source localization algorithm employed. Our analyses revealed that the collection of EEG data using a high-density montage is crucial for RSN detection by sICA, but also the use of appropriate methods for head modeling and source localization have a substantial effect on RSN reconstruction. Overall, our results confirm the potential of hdEEG for mapping the functional architecture of the human brain, and highlight at the same time the interplay between acquisition technology and innovative solutions in data analysis.

## Introduction

Functional interactions within large-scale networks of neuronal assemblies can be quantified by *functional connectivity* (FC) methods, which estimate the statistical dependence between dynamic activity recorded from distinct brain areas (Friston, 2011). FC is most commonly measured from functional magnetic resonance imaging (fMRI) data, which have spatial resolution of the order of some millimeters and permit to reliably map large-scale functional networks across the brain (Fox and Raichle, 2007; Gillebert and Mantini, 2013). FC analysis of fMRI signals has seen a tremendous rise of popularity during the last years, as it provides an effective and easy-to-apply tool for studying both healthy and diseased brains (van den Heuvel and Hulshoff Pol, 2010; Gillebert and Mantini, 2013). Electroencephalography (EEG) or magnetoencephalography (MEG) may be utilized in alternative to fMRI to examine functional interactions within large-scale brain networks (Ganzetti and Mantini, 2013; Marzetti et al., 2013; Brookes et al., 2014, 2016; Florin and Baillet, 2015; Tewarie et al., 2016). Despite a number of technical issues primarily due to the fact that these techniques yield signals measured from outside the head, they are potentially more suited than fMRI to investigate long-range neuronal communication at higher temporal resolution (de Pasquale et al., 2010, 2012, 2016, 2017; Baker et al., 2014; Vidaurre et al., 2016; O’Neill et al., 2017).

Notably, the applications of EEG in the context of FC are potentially superior compared to MEG, mainly because the EEG equipment is portable, has low maintenance costs and can be used in combination with other brain imaging and stimulation techniques (Michel and Murray, 2012). However, it should be considered that source localization with EEG is often more challenging than with MEG. In fact, EEG-based source localization requires the use of precise, realistic biophysical models that incorporate the exact positions of the sensors as well as the conductivity properties of the head tissues. Furthermore, source estimation with EEG, but also with MEG, is underspecified in nature yielding a blurred image of the true activity at the voxel level, due to the ill-posedness of inverse solutions (Grech et al., 2008). This issue is partially addressed by constraining the sources into the volume conductor. However, given the fact that brain activity is estimated from a finite number of recordings, spurious correlations between reconstructed timecourses of neighboring voxels are present (Hillebrand et al., 2012). Such an effect is referred to as “signal leakage” (Brookes et al., 2012; Hipp et al., 2012; Colclough et al., 2015). In the case of EEG, the signal leakage problem is largely dependent on the spatial sampling density and coverage of the electrode montage (Slutzky et al., 2010; Song et al., 2015). From this standpoint, the use of high-density EEG (hdEEG) which provides both high spatial sampling density and large head coverage, may facilitate brain activity reconstruction (Lantz et al., 2003) and FC analyses (Liu et al., 2017).

To mitigate the effect of signal leakage in the identification of EEG networks, we have recently proposed the use of independent component analysis (ICA) (Liu et al., 2017). ICA performs a blind decomposition of a given number of spatio-temporal patterns that are mixed in the data, assuming that these patterns are mutually and statistically independent in time (temporal ICA, tICA) or space [spatial ICA, spatial independent component analysis (sICA)]. It yields a number of independent components (ICs), each of which consists of a spatial map and an associated time-course (Calhoun et al., 2001). The IC spatial map reveals brain regions that have a similar response pattern, and are therefore considered to be functionally connected (Mantini et al., 2007; Brookes et al., 2011). For resting state fMRI studies, sICA has been widely used as it permits to map multiple resting state networks (RSNs) in a data-driven fashion, whereas the applications of tICA in the context of fMRI remain limited (McKeown et al., 1998; Smith et al., 2012). In our previous work, we have shown that both tICA and sICA permit the detection of RSNs from hdEEG data. Interestingly, the EEG-RSNs obtained with sICA were remarkably similar to fMRI-RSNs derived using the same connectivity approach. This finding supports the idea that hdEEG can be a novel tool for mapping the functional architecture of the human brain in health and disease. Before exploiting the utility of hdEEG in the context of brain network analysis, however, it is important to clarify what technological aspects of hdEEG acquisition and analysis primarily influence the accurate detection of EEG-RSNs by sICA. In this study, we examine to what extent the reconstruction of EEG-RSN maps depends on the electrode density and coverage, the accuracy of the head model, and the source localization algorithm employed.

## Materials and Methods

### Subjects and Data

Data used in this study comprise resting-state hdEEG signals, electrode positions and individual whole-head anatomy MRI, which were collected in 19 healthy right-handed subjects (age 28 ± 5.9 years, 5 males and 14 females) following experimental procedures approved by the local Institutional Ethics Committee of ETH Zürich. In short, high-density EEG (hdEEG) signals were recorded for 5 min at 1000 Hz by using a 256-channel system from Electrical Geodesics (EGI, Eugene, OR, United States). Horizontal and vertical electrooculographic (hEOG/vEOG) and electromyographic signals (EMG) were recorded as well. During data collection, participants were fixating a black cross in the center of a white screen. Prior to EEG acquisition, sensors position coordinates were obtained by using a Geodesic Photogrammetry System (GPS) (Russell et al., 2005). A T1-weighted whole-head MR image of each subject was acquired in a separate experimental session using a Philips 3T Ingenia scanner with a turbo field echo sequence. The scanning parameters were: TR = 8.25 ms, TE = 3.8 ms, flip angle = 8°, 160 sagittal slices, matrix size = 240 × 240, voxel size = 1 mm^3^. The total acquisition time was around 6 min.

### Workflow for EEG Network Detection

**FIGURE 1:**
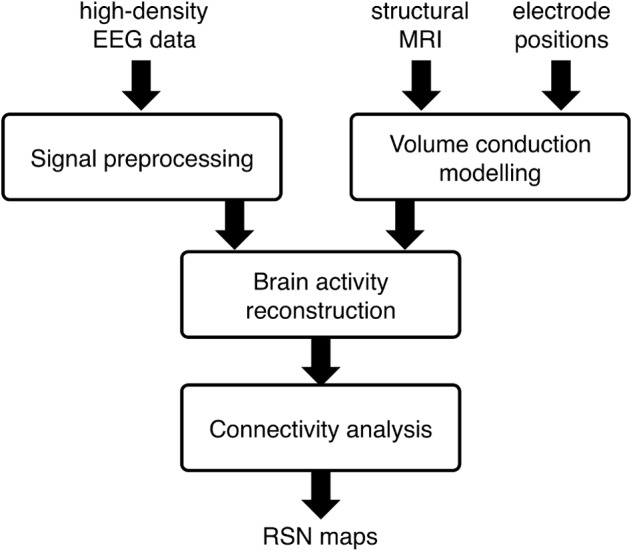
Workflow for the spatial independent component analysis (sICA)-based network analysis using high-density electroencephalography (hdEEG) data. The main analysis steps include: (1) *Signal preprocessing*, to attenuate noise and artifacts that are mixed in the EEG data; (2) *Volume conduction modeling*, to establish how brain sources can generate specific distributions of EEG potentials; (3) *Brain activity reconstruction*, to estimate -based on the clean EEG data and the volume conduction model- the distribution of active brain sources that most likely generates the EEG potentials; (4) *Connectivity analysis*, to obtain RSN maps by using sICA on source-space power envelopes.

Our analysis workflow for EEG-RSN detection using sICA was introduced in our previous work (Liu et al., 2017). Four main analysis steps are involved (see **Figure 1**): (1) *Signal preprocessing*, to attenuate noise and artifacts that are mixed in the EEG data; (2) *Volume conduction modeling*, to establish how brain sources can generate specific distributions of EEG potentials; (3) *Brain activity reconstruction*, to estimate -based on the clean EEG data and the volume conduction model- the distribution of active brain sources that most likely generates the EEG potentials; (4) *Connectivity analysis*, to obtain RSN maps by using sICA on source-space power envelopes.

#### Signal Preprocessing

First, we used an automated procedure to detect channels with low signal quality based on the minimum Pearson correlation of the signal against all the signals from the other channels, as well as on the noise variance. The number of detected channels with low signal quality ranged between 1 and 8, depending on the specific dataset. The signals from those channels were interpolated based on signals from the neighboring channels, using the FieldTrip toolbox^1^. Accordingly, all EEG datasets later used for source localization had the same number of signals. Subsequently, we band-pass filtered the EEG data in the frequency range 1–80 Hz and we decomposed them into ICs by using the fast fixed-point ICA (FastICA) algorithm^2^, to identify and remove artifacts of biological origin (Mantini et al., 2008). Artifactual ICs were automatically identified by using information from the signal kurtosis, the power spectrum and the correlation with horizontal and vertical electrooculogram (hEOG and vEOG) and electromyogram (EMG). Finally, we re-referenced the cleaned EEG signals using the average reference approach (Liu et al., 2015).

#### Volume Conduction Modeling

First of all, we performed a segmentation of the individual head in the MR image in 12 different tissue classes (skin, eyes, muscle, fat, spongy bone, compact bone, cortical gray matter, cerebellar gray matter, cortical white matter, cerebellar white matter, cerebrospinal fluid and brain stem) by means of SPM12^3^. This segmentation was based on template tissue classes from the MIDA model (Iacono et al., 2015). We then spatially coregistered the EEG electrodes to the skin compartment in the MR space using the Iterative Closest Point registration algorithm (Besl and Mckay, 1992) implemented in SPM12. After associating each tissue class with a representative conductivity value (Haueisen et al., 1997) (see **Supplementary Table S1**), we used the SimBio finite element method (FEM) integrated in FieldTrip for the numerical approximation of the volume conduction model (Wolters et al., 2004). Alternatively to the SimBio-FEM, a boundary element method (BEM) solution can be used in our analysis workflow for the creation of the volume conduction model. When using BEM, the tissue classes need to be encapsulated, and their number is typically restricted to three or four (Gencer and Acar, 2004). The dipoles corresponding to potential brain sources were placed on a regular 6-mm grid spanning the cortical gray matter and cerebellar gray matter. The number of dipoles ranged between 2877 and 3354, depending on the specific dataset. The orientation of the dipoles was free, to possibly account for the relatively large volume covered by the dipole itself. Finally, a leadfield matrix expressing the scalp potentials corresponding to each single-dipole source configuration was generated based on the volume conduction model.

#### Brain Activity Reconstruction

For the reconstruction of brain activity, we integrated the information from the artifact-free hdEEG recordings and the leadfield matrix. We implemented several source localization algorithms in our analysis workflow, among which the minimum norm estimator (MNE), the linear constraint minimum variance beamformer (LCMV), the standardized low-resolution brain electromagnetic tomography (sLORETA) and the exact low-resolution brain electromagnetic tomography (eLORETA) algorithms (Pascual-Marqui et al., 2011). In the MNE implementation, noise prewhitening of the leadfield matrix was applied using the noise covariance matrix, with regularization parameter λ = 0.1. In addition, the signal to noise ratio was set equal to 5 and the depth weighting to 0.5. In the LCMV implementation, the Tikhonov approach was used to set the regularization parameter. As for sLORETA, the signal to noise ratio was set equal to 5 and no depth weighting was applied.

Based on previous tests, eLORETA was chosen as the default source localization algorithm in our analysis pipeline. After reconstructing neuronal activity at each cortical voxel, we calculated power time-courses on the whole frequency band under investigation (1–80 Hz). We used a moving window with 1-s duration to enhance detection of co-modulations across distant brain regions, as done in previous MEG and EEG studies (de Pasquale et al., 2010; Liu et al., 2017).

#### Connectivity Analysis

The detection of RSNs based on the reconstructed power timecourses was performed using sICA (Bartels and Zeki, 2005). The number of ICs was estimated by using the minimum description length (MDL) criterion (Li et al., 2007). This number ranged between 34 and 58, depending on the specific EEG dataset. ICA decomposition was performed using the FastICA algorithm, which was run 10 times using a deflation approach and hyperbolic tangent as contrast function^4^ (Himberg and Hyvarinen, 2003). EEG-RSNs of interest were selected by using a template-matching procedure. Specifically, the templates were warped to individual MR space. Then, the Pearson correlation was used to estimate the similarity in the spatial distribution of the EEG-ICs and the template RSN maps. The best match of EEG-IC for each template map was extracted iteratively, labeled as a specific EEG-RSN, and removed from the pool of EEG-ICs. This impeded that the same IC was associated with two different templates.

### Assessment of EEG-RSN Detection Performance

We assessed EEG-RSN detection performance on the basis of fMRI-RSN maps derived from data used in one of our previous studies (Mantini et al., 2013). Ethical approval was granted by the Ethics Committee of Chieti University. The experiment was performed in accordance with the relevant guidelines and regulations, and informed consent was obtained from all participants. The fMRI data, which were collected in 24 healthy young subjects at rest for 10 min. Brain networks were detected using sICA from each individual fMRI dataset, as done for hdEEG data. Group-level IC maps were obtained using the self organized clustering ICA (sog-ICA) method (Mantini et al., 2013). fMRI-RSN were extracted from the set of group-level ICs, on the basis of their spatial map. Our investigations were focused on six core brain networks that were robustly found in previous fMRI studies (Bartels and Zeki, 2005; Mantini et al., 2007): default mode network (DMN), dorsal attention network (DAN), visual foveal network (VFN), auditory network (AN), dorsal somatomotor network (DSN), and medial prefrontal network (MPN). We used 5000 permutations for the across-subject fMRI-RSN analysis, and we set the significance threshold to *p* < 0.01 corrected for multiple comparisons by using the threshold-free cluster enhancement (TFCE) method (Smith and Nichols, 2009).

We performed EEG-RSN detection on each hdEEG datasets, following our default analysis strategy and using the fMRI maps as templates (see **Supplementary Figure S1**). For each EEG-RSN, we transformed the individual maps to common space using SPM and derived a group-level RSN map by using the same statistical approach applied to fMRI data. The spatial correspondence of the EEG networks with the fMRI networks was quantified using the Pearson correlation between maps. To verify that the detected EEG-RSNs were selectively associated with a specific fMRI-RSN, we calculated a matrix of cross-correlations between EEG-RSN and fMRI-RSN maps. Moreover, we tested the robustness of EEG-RSN spatial patterns obtained by sICA by performing a split-half analysis. Specifically, we split the 5-min recording into two segments of equal duration. We independently obtained EEG-RSNs from each of these two data segments, and we calculated the Pearson correlation between them.

As a further analysis step, we investigated the impact of the number of EEG channels, the accuracy of the head model and source localization algorithm on EEG-RSN detection by sICA. To investigate the RSNs with lower montage density, we spatially subsampled each set of 256-channel recordings and derived 32-channel, 64-channel and 128-channel recordings with electrodes positioned according to standard EEG montages. The effect of using a less accurate head model was tested by running RSN detection on source data reconstructed using a 5-layer realistic FEM, and a 3-layer BEM based either on an individual or a template MR image. In line with previous literature (Ramon et al., 2006; Wolters et al., 2006; Cho et al., 2015), the 5-layer FEM model comprised gray matter (cortical and cerebellar), white matter (cortical and cerebellar) plus brainstem, cerebrospinal fluid, skull (compact and spongy) and all remaining soft tissues (skin, eyes, muscles, and fat). In turn, the 3-layer BEM models included brain, skull and all other tissues. We obtained the conductivity values for 5- and 3-layer models by pooling together different tissues and averaging the conductivity values used for the 12-layer head model. Finally, we examined whether it was possible to detect EEG-RSNs using different source localization methods for the reconstruction of brain activity in the source space. To this end, we tested the RSN results obtained using eLORETA against those obtained with sLORETA, MNE, and LCMV. EEG-RSN reconstruction performance with different number of EEG channels, head model and source localization algorithm was quantified by calculating the spatial correlation with the EEG-RSNs that were obtained following our default analysis settings (i.e., 256-channel montage, 12-layer FEM, eLORETA source localization), as well as the fMRI RSNs. Significant effects of electrode montage, head modeling and source localization, respectively, were assessed by means of a repeated-measure analysis of variance (ANOVA), calculated on Fisher-transformed correlation values.

## Results

### Comparison Between EEG-RSNs and fMRI-RSNs

**FIGURE 2:**
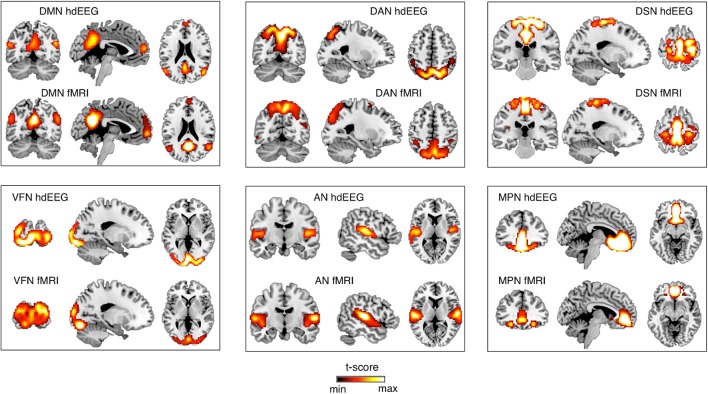
Comparison between EEG-RSN and fMRI-RSN maps. EEG-RSN maps were obtained by applying sICA on 5-min source-space power envelopes (*N* = 19, threshold: *p* < 0.01, TFCE corrected). They were spatially compared with RSNs maps obtained by applying sICA on 10-min fMRI data (*N* = 24, threshold: *p* < 0.01, TFCE corrected). DMN, default mode network; DAN, dorsal attention network; DSN, dorsal somatomotor network; VFN, visual foveal network; AN, auditory network; MPN, medial prefrontal network.

**FIGURE 3:**
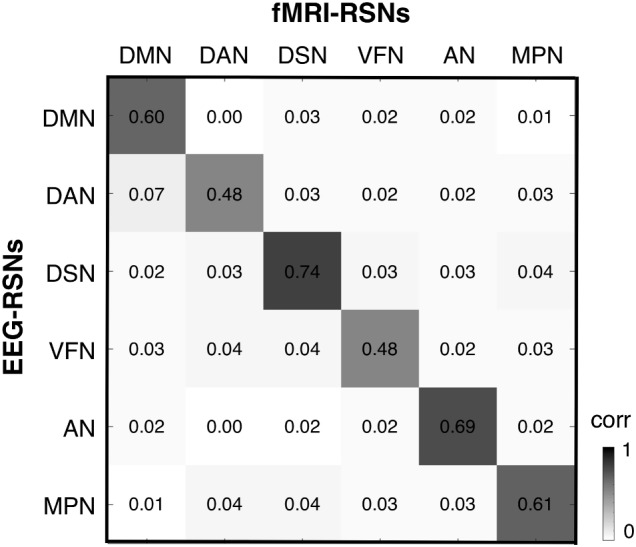
Spatial correspondence between EEG-RSN and fMRI-RSN maps. We calculated a cross-correlation matrix, showing the spatial similarity between any pair of EEG- and fMRI-RSN. DMN, default mode network; DAN, dorsal attention network; DSN, dorsal somatomotor network; VFN, visual foveal network; AN, auditory network; MPN, medial prefrontal network.

**FIGURE 4:**
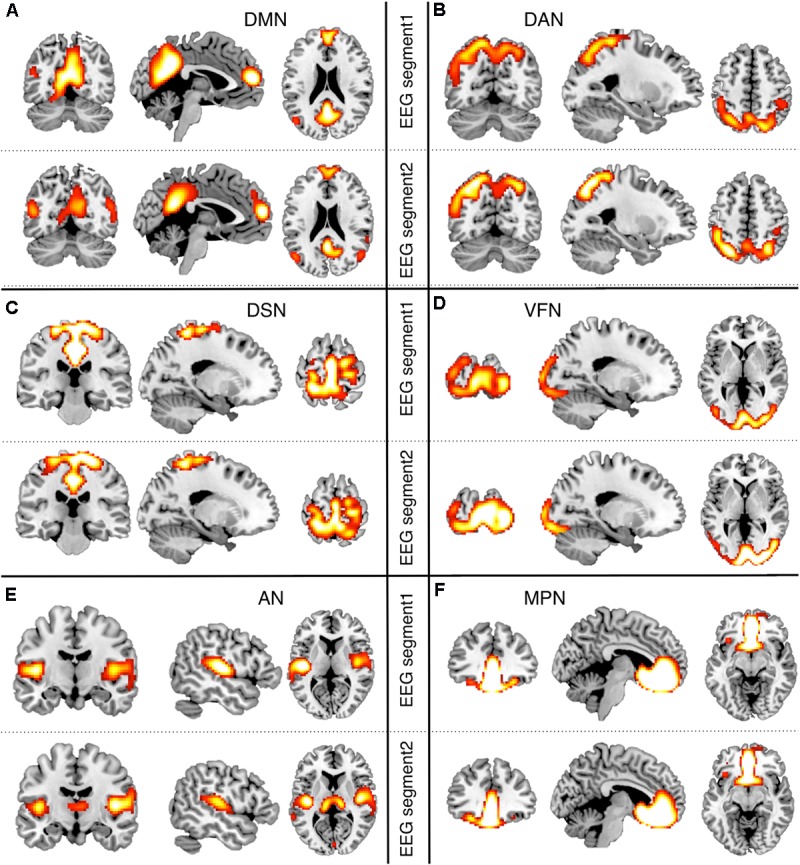
Split-half analysis of EEG-RSNs. We split the hdEEG recordings into two segments: the first 2 min and 30 s (EEG segment 1) and the last 2 min and 30 s (EEG segment 2). RSN maps obtained from the two EEG segments were directly compared (threshold: *p* < 0.01, TFCE corrected). **(A)** DMN, default mode network; **(B)** DAN, dorsal attention network; **(C)** DSN, dorsal somatomotor network; **(D)** VFN, visual foveal network; **(E)** AN, auditory network; **(F)** MPN, medial prefrontal network.

After obtaining EEG-RSNs using our analysis workflow, we compared them with the corresponding RSNs detected from fMRI data (**Figures 2**, **3**). This comparison revealed that a complete network topology could be reconstructed by using hdEEG data for DMN, VFN, AN, DSN, MPN, but not DAN. For this latter RSN, the spatial map did not include frontal eye field areas, which were below the statistical threshold. Overall, the EEG-RSNs were found to be remarkably similar to the corresponding fMRI-RSNs. Specifically, the correlations between group-level EEG- and fMRI-RSNs were remarkable (0.39 ≤ *r* ≤ 0.67, $\overset{-}{r}$ = 0.57). The robustness of EEG-RSN detection was confirmed by the split-half analysis (**Figure 4**). In particular, the RSN maps obtained from two different segments of hdEEG data were largely matching (0.62 ≤ *r* ≤ 0.79, $\overset{-}{r}$ = 0.70).

### Impact of Montage Density, Head Modeling and Source Localization

**FIGURE 5:**
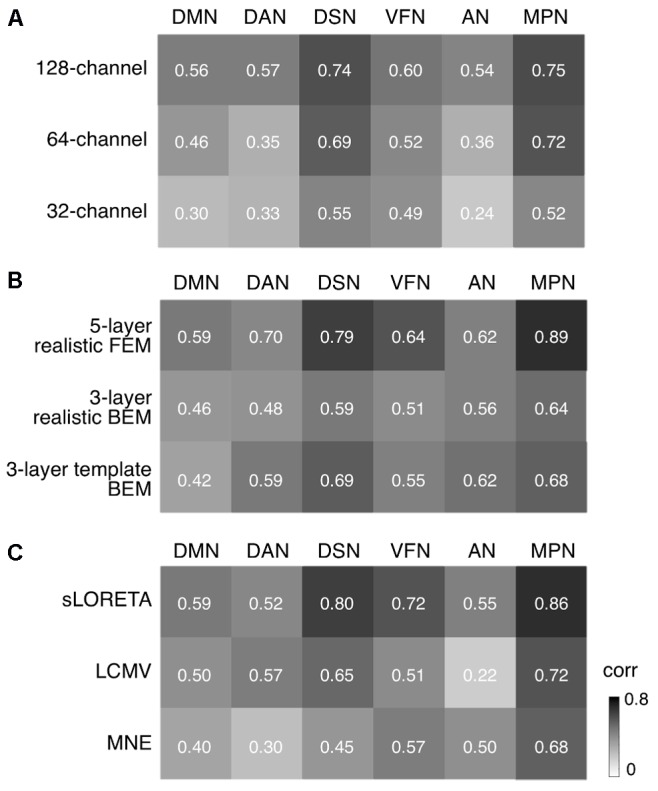
Impact of EEG montage density, head modeling and source localization on RSN reconstruction. We defined the EEG-RSNs obtained with default settings as reference, and calculated their spatial correlation with the corresponding RSNs calculated using different settings. **(A)** We examined the impact of EEG montage density by comparing the maps obtained from 256-channel recordings with those obtained from 128-, 64-, and 32-channel recordings. **(B)** We examined the impact of head modeling by comparing the maps obtained by using a 12-layer FEM with those obtained by 5-layer realistic FEM, 3-layer realistic BEM and 3-layer template BEM, respectively. **(C)** We examined the impact of source localization by comparing the maps for eLORETA with those obtained by sLORETA, LCMV, and MNE, respectively. DMN, default mode network; DAN, dorsal attention network; DSN, dorsal somatomotor network; VFN, visual foveal network; AN, auditory network; MPN, medial prefrontal network.

**FIGURE 6:**
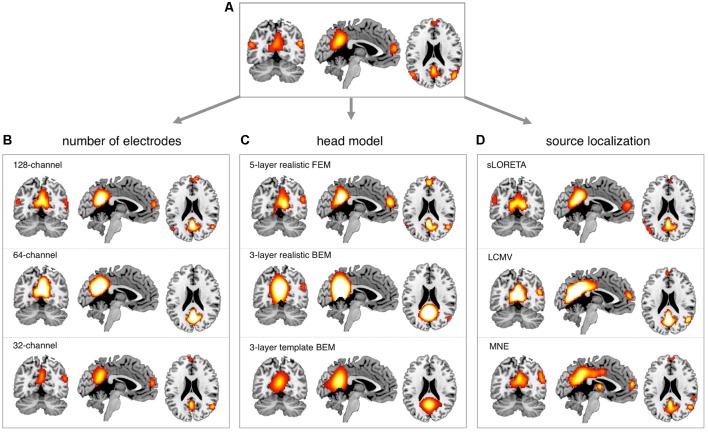
Impact of EEG montage density, head modeling and source localization on DMN reconstruction. EEG-RSNs were obtained: **(A)** using 256-channel recordings, 12-layer realistic FEM and eLORETA source localization. **(B)** Using 128-, 64-, and 32-channel recordings, respectively, with 12-layer realistic FEM and eLORETA source localization. **(C)** Using 5-layer realistic FEM, 3-layer realistic BEM and 3-layer template BEM, respectively, with 256-channel recordings and eLORETA source localization. **(D)** Using sLORETA, LCMV and MNE localization, respectively, with 256-channel recordings and 12-layer realistic FEM.

**FIGURE 7:**
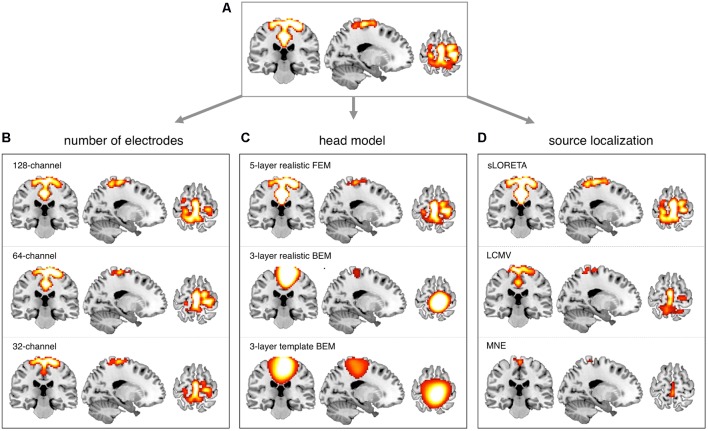
Influence of EEG montage density, head modeling and source localization on DSN reconstruction. EEG-RSNs were obtained: **(A)** using 256-channel recordings, 12-layer realistic FEM and eLORETA source localization. **(B)** Using 128-, 64-, and 32-channel recordings, respectively, with 12-layer realistic FEM and eLORETA source localization. **(C)** Using 5-layer realistic FEM, 3-layer realistic BEM and 3-layer template BEM, respectively, with 256-channel recordings and eLORETA source localization. **(D)** Using sLORETA, LCMV, and MNE localization, respectively, with 256-channel recordings and 12-layer realistic FEM.

We conducted more detailed analyses to better understand to what extent the sICA results depended on specific hdEEG analysis aspects. To this end, we analyzed the impact of montage density, head modeling and source localization (**Figures 5**–**7** and **Supplementary Figures S2**–**S6**). By using a repeated-measure ANOVA, we revealed that all these three aspects are important for EEG-RSN reconstruction, with montage density potentially having the strongest effects (*F* = 29.26, *p* < 0.0001), followed by head modeling (*F* = 11.12, *p* = 0.0029) and source localization (*F* = 7.99, *p* = 0.0085). When we examined in detail the influence of the montage density, we found that RSNs comprising deeper brain regions, such as the DMN, were more affected by a reduced number of EEG channels (**Figure 6**). Conversely, minimal differences were observed for the DSN with different EEG montage density (**Figure 7**). Also, our analyses confirmed the importance of using an accurate head model. For some networks, as for instance the DSN, it was relatively difficult to appreciate differences between the results of a 12-layer FEM, which was our default solution, with those of a 5-layer FEM (**Figure 7**). Conversely, there was a substantial mismatch with the RSNs obtained by using a 3-layer BEM, built either on an individual or a template MR image (**Figures 5B**, **6**, **7**). Also, we found that the RSNs with our default solution for source localization, i.e., the eLORETA method, was only slightly different from those obtained by using sLORETA. In contrast, the reconstruction improvement as compared to MNE and LCMV methods was remarkable (**Figures 5C**, **6**, **7**).

## Discussion

In this study we sought to determine to what extent EEG-RSNs that spatially correspond to fMRI-RSNs can be reliably detected by means of sICA, and how the accuracy of the EEG-RSN reconstructions is influenced by the EEG montage density, as well as the specific solutions adopted for head modeling and source localization. Importantly, our results confirmed the similarity of EEG-RSNs with fMRI-RSNs obtained by means of sICA. Also, they revealed that the collection of EEG data using a high-density montage is crucial for RSN detection, and that the use of specific methods for head modeling and source localization are also important to ensure accurate network reconstructions.

In our recent work (Liu et al., 2015, 2017; Marino et al., 2016), we have proposed technological solutions enabling the use of hdEEG for the study of neuronal dynamics in the human brain. Currently available hdEEG systems have at most 256 recording channels, which is a number comparable to that of MEG systems (Brookes et al., 2011; Hipp et al., 2012). We reasoned that the use of a 256-dimensional hdEEG dataset might be theoretically sufficient to resolve the activity of functionally different areas in the brain, which are thought to be around 200 (Glasser et al., 2016). In this study, we were particularly interested in examining if EEG-RSNs could be detected using sICA with 32-, 64-, and 128-channel EEG recordings, and to what extent these networks were matching the ones reconstructed with 256-channel EEG recordings. Overall, our results strongly emphasized the importance of using high-density montages for the EEG-RSN studies (**Figures 5A**, **6**, **7**). This was especially the case for RSNs including distant brain regions such as the DMN (**Figure 6**); however, RSNs with a less distributed pattern, as for instance the DSN, could be successfully reconstructed also with a low density EEG montage (**Figure 7**).

Achieving an accurate reconstruction of neuronal activity with hdEEG is undoubtedly more difficult than with MEG, as it depends on the availability of precise biophysical models relating the spatial configuration of currents in the cortex with potentials measured over the scalp (Michel et al., 2004; Michel and Murray, 2012). The generation of a realistic head model is one of the main challenges for hdEEG analyses at the source level, which we addressed by integrating in our analysis workflow a 12-layer FEM for volume conduction modeling. Surprisingly, the results of our study suggested that the use of less precise head models has a relatively smaller impact on EEG-RSN reconstruction than poorer spatial sampling and coverage of EEG electrodes over the scalp (**Figures 5B**, **6**, **7**). We noticed that more accurate EEG-RSN maps could be reconstructed using a head model created with a 12-layer FEM as compared to 5-layer FEM. The improvement was, however, much more marked compared to 3-layer BEMs, in which white matter and gray matter belong to the same layer and to which the same conductivity value is assigned (Gencer and Acar, 2004).

In our analysis workflow, eLORETA is the standard source localization algorithm for the reconstruction of ongoing brain activity. It is worth noting that the performance of source localization algorithms depends on the source depth, the noise level, the number of recording electrodes and the head model (Michel et al., 2004), and there is no general consensus about which source localization method delivers best performance for EEG (Michel et al., 2004). Our results confirmed the suitability of eLORETA, but also sLORETA, for EEG connectivity investigations (**Figures 5C**, **6**, **7**). On the other hand, the RSNs reconstructed using MNE and LCMV were relatively less similar to fMRI-RSNs. eLORETA has already been shown to be particularly accurate in the presence of low-noise signals (Pascual-Marqui et al., 2011) and has been already tested in the context of EEG connectivity (Aoki et al., 2015; Bachinger et al., 2017; Liu et al., 2017). Overall, our quantitative analyses on the correspondence between EEG-RSNs and fMRI-RSNs (**Figures 2**, **3**) lend support to our choice in terms of head modeling and source localization methods.

Our study disclosed important information concerning the robustness of EEG-RSNs detected by sICA and their spatial similarity to fMRI-RSNs. A number of limitations should, however, be mentioned. First of all, EEG-RSN detection performance was examined when using several methods for head modeling and source localization, which were selected among the most commonly used for EEG analysis. Testing a larger number of head modeling and source localization methods was not possible for computational reasons. It should be mentioned that source localization methods are particularly sensitive to their specific input parameters. We intentionally used standard settings for these methods. It is, however, conceivable that superior source localization performance, and therefore better EEG-RSN reconstruction, could be achieved by optimizing input parameters for any given method. Also, hdEEG data were collected in each participant during a single acquisition session. This means that we could only perform split-half analyses to assess the reproducibility of EEG-RSN detection, whereas we could not carry out test–retest analyses. Furthermore, the EEG- and fMRI-RSN maps being compared were obtained from different groups of participants. As such, spatial similarities could be assessed only at the group level, and not subject-by-subject. In the future, it would be interesting to examine the spatial correspondence of EEG- and fMRI-RSNs within the same subjects, possibly using simultaneous EEG-fMRI recordings. Previous studies showed the potential of simultaneous EEG-fMRI to elucidate the neural correlates of FC in brain networks (Mantini et al., 2007; Lei et al., 2011, 2014).

In summary, we performed an extensive validation concerning the use of sICA for the detection of RSNs using hdEEG recordings. We observed a remarkable similarity of EEG-RSNs with fMRI-RSNs. A split-half analysis confirmed the robustness of EEG-RSN detection, even with short EEG recordings. Also, we showed the sensitivity of EEG-RSN detection to the use of different electrode montages, head models and source localization methods. Ultimately, our results confirm the potential of hdEEG for mapping the functional architecture of the human brain, and highlight at the same time the interplay between acquisition technology and innovative solutions in data analysis.

## Ethics Statement

EEG and fMRI data collection was approved by the Ethics Commission of ETH Zürich and Chieti University, respectively. All participants signed a written informed consent.

## Author Contributions

DM and NW designed the research. QL and MG produced the results. QL and DM wrote the manuscript, which was read and approved by the other co-authors.

## Conflict of Interest Statement

The authors declare that the research was conducted in the absence of any commercial or financial relationships that could be construed as a potential conflict of interest.

## Footnotes

1 http://www.fieldtriptoolbox.org

2 http://research.ics.aalto.fi/ica/fastica

3 http://www.fil.ion.ucl.ac.uk/spm/software/spm12

4 http://research.ics.aalto.fi/ica/icasso

## Supplementary Material

FIGURE S1 Six fMRI-RSNs maps used as templates in the study. The maps were obtained using sICA, by reanalyzing previously published resting state fMRI data from 24 healthy subjects (Mantini et al., 2013).

FIGURE S2 Impact of the number of EEG channels, the accuracy of the head model and source localization algorithm on EEG-RSN detection. We defined the fMRI-RSNs as reference, and calculated the spatial correlation with the corresponding EEG-RSNs calculated using different settings. In each case, the spatial correlation values were divided by those of EEG RSNs obtained using default settings (i.e., 256-channel recordings, 12-layer FEM for head modeling and eLORETA for source localization). **(A)** We examined the impact of EEG montage density by comparing the maps obtained from 128-, 64-, and 32-channel recordings, with respect to 256-channel recordings. **(B)** The impact of head modeling by comparing the maps obtained by 5-layer realistic FEM, 3-layer realistic boundary element model (BEM) and 3-layer template BEM, with respect to a 12-layer FEM. **(C)** The impact of source localization by comparing the maps obtained by sLORETA, LCMV, and MNE, with respect to eLORETA.

FIGURE S3 Influence of EEG montage density, head modeling and source localization on DAN reconstruction. EEG-RSNs were obtained: **(A)** using 256-channel recordings, 12-layer realistic FEM and eLORETA source localization. **(B)** Using 128-, 64-, and 32-channel recordings, respectively, with 12-layer realistic FEM and eLORETA source localization. **(C)** Using 5-layer realistic FEM, 3-layer realistic BEM and 3-layer template BEM, respectively, with 256-channel recordings and eLORETA source localization. **(D)** Using sLORETA, LCMV and MNE localization, respectively, with 256-channel recordings and 12-layer realistic FEM.

FIGURE S4 Influence of EEG montage density, head modeling and source localization on VFN reconstruction. EEG-RSNs were obtained: **(A)** using 256-channel recordings, 12-layer realistic FEM and eLORETA source localization. **(B)** Using 128-, 64- and 32-channel recordings, respectively, with 12-layer realistic FEM and eLORETA source localization. **(C)** Using 5-layer realistic FEM, 3-layer realistic BEM and 3-layer template BEM, respectively, with 256-channel recordings and eLORETA source localization. **(D)** Using sLORETA, LCMV, and MNE localization, respectively, with 256-channel recordings and 12-layer realistic FEM.

FIGURE S5 Influence of EEG montage density, head modeling and source localization on AN reconstruction. EEG-RSNs were obtained: **(A)** using 256-channel recordings, 12-layer realistic FEM and eLORETA source localization. **(B)** Using 128-, 64-, and 32-channel recordings, respectively, with 12-layer realistic FEM and eLORETA source localization. **(C)** Using 5-layer realistic FEM, 3-layer realistic BEM and 3-layer template BEM, respectively, with 256-channel recordings and eLORETA source localization. **(D)** Using sLORETA, LCMV, and MNE localization, respectively, with 256-channel recordings and 12-layer realistic FEM.

FIGURE S6 Influence of EEG montage density, head modeling and source localization on MPN reconstruction. EEG-RSNs were obtained: **(A)** using 256-channel recordings, 12-layer realistic FEM and eLORETA source localization. **(B)** using 128-, 64-, and 32-channel recordings, respectively, with 12-layer realistic FEM and eLORETA source localization. **(C)** Using 5-layer realistic FEM, 3-layer realistic BEM and 3-layer template BEM, respectively, with 256-channel recordings and eLORETA source localization. **(D)** Using sLORETA, LCMV and MNE localization, respectively, with 256-channel recordings and 12-layer realistic FEM.

TABLE S1 Conductivity values of different tissues used for the calculation of the head model. The conductivity values associated with the tissue classes were extracted from relevant literature (Haueisen et al., 1997).
