# Supplementary figures and images for: Detecting Large-Scale Brain Networks Using EEG: Impact of Electrode Density, Head Modeling and Source Localization
Source: Front Neuroinform. 2018 Mar 2;12:4. doi: 10.3389/fninf.2018.00004 (PMC5841019; doi:10.3389/fninf.2018.00004)

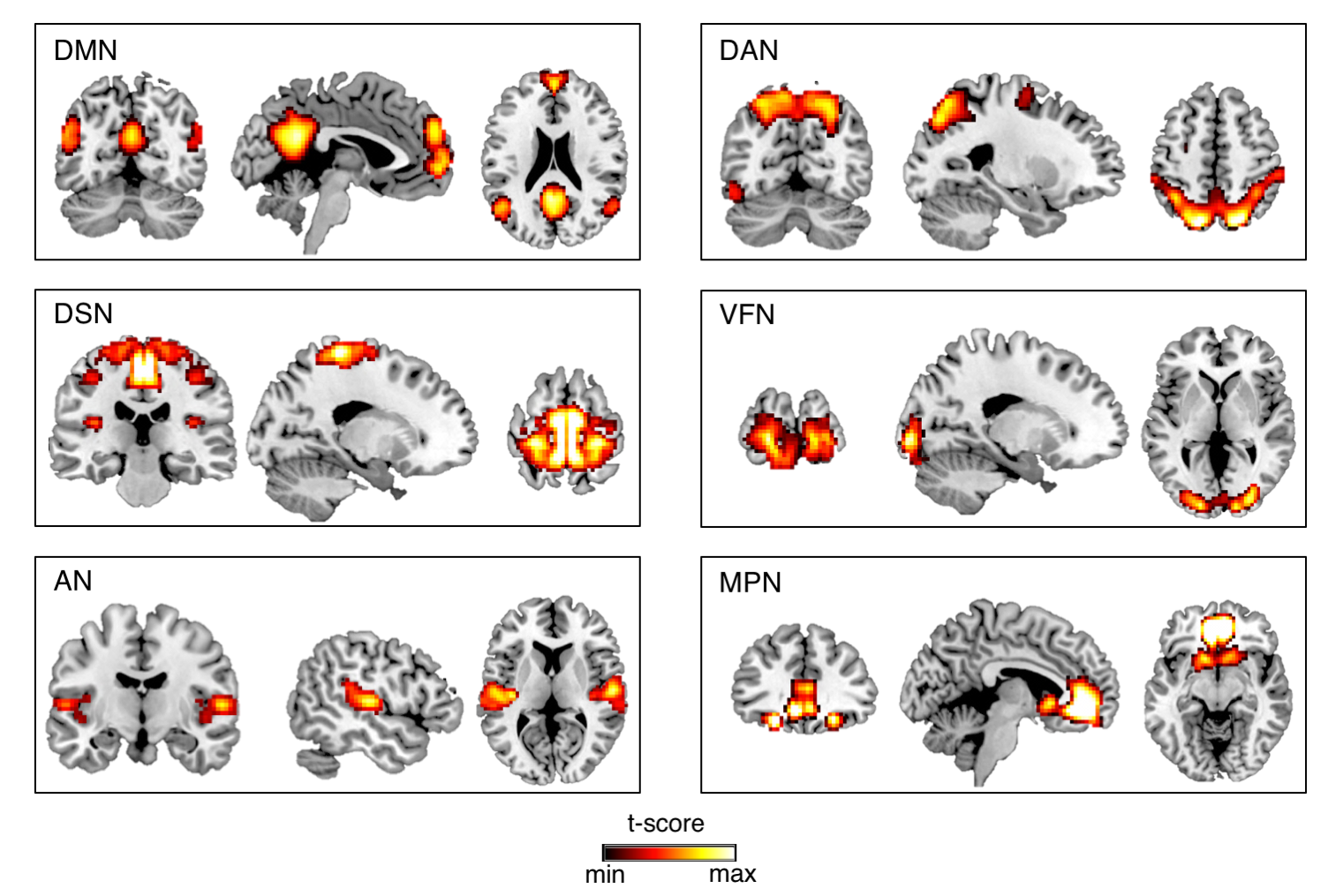

Supplement: FIGURE S1 — Six fMRI-RSNs maps used as templates in the study. The maps were obtained using sICA, by reanalyzing previously published resting state fMRI data from 24 healthy subjects (Mantini et al., 2013). [file Image_1.TIFF]

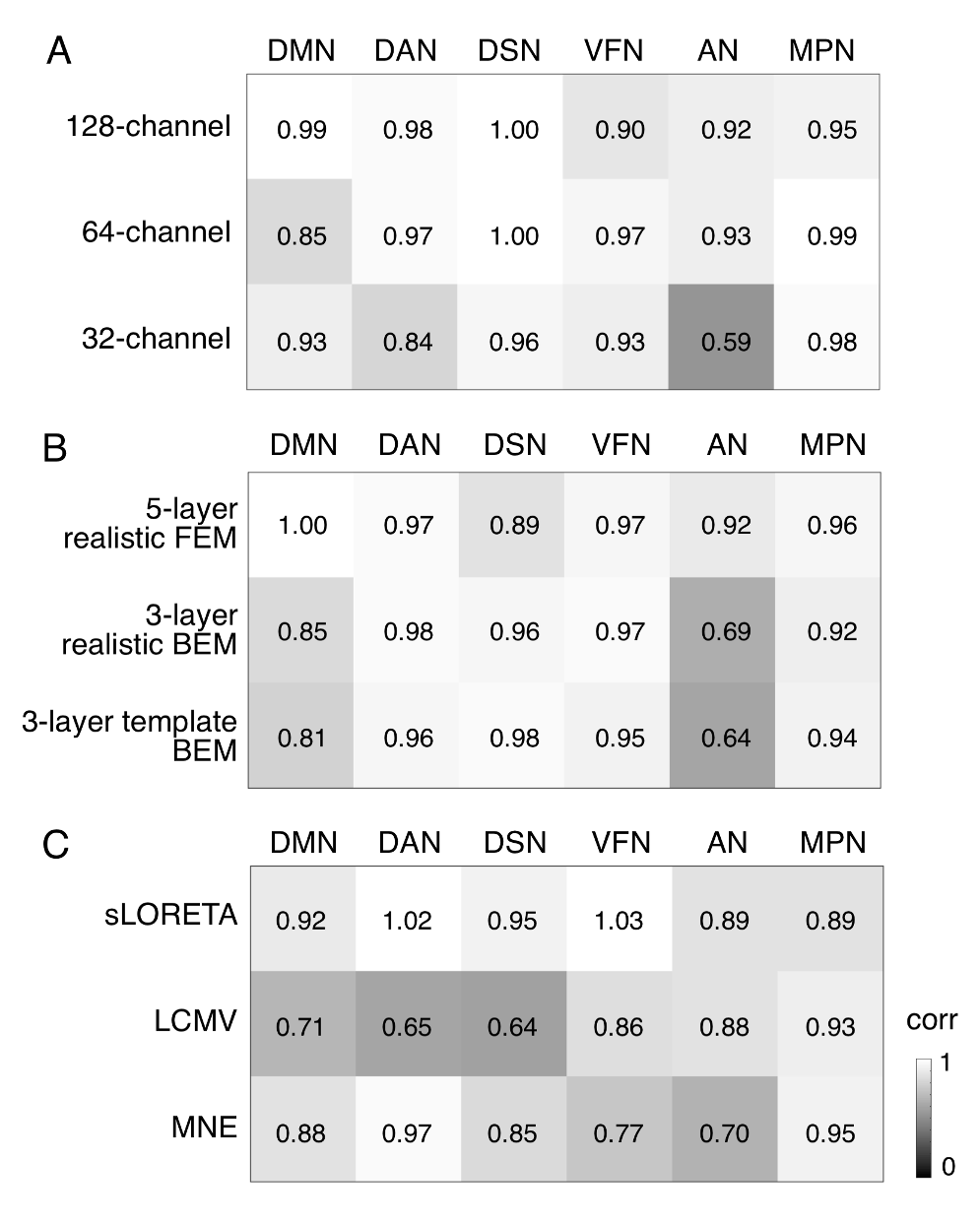

Supplement: FIGURE S2 — Impact of the number of EEG channels, the accuracy of the head model and source localization algorithm on EEG-RSN detection. We defined the fMRI-RSNs as reference, and calculated the spatial correlation with the corresponding EEG-RSNs calculated using different settings. In each case, the spatial correlation values were divided by those of EEG RSNs obtained using default settings (i.e., 256-channel recordings, 12-layer FEM for head modeling and eLORETA for source localization). (A) We examined the impact of EEG montage density by comparing the maps obtained from 128-, 64-, and 32-channel recordings, with respect to 256-channel recordings. (B) The impact of head modeling by comparing the maps obtained by 5-layer realistic FEM, 3-layer realistic boundary element model (BEM) and 3-layer template BEM, with respect to a 12-layer FEM. (C) The impact of source localization by comparing the maps obtained by sLORETA, LCMV, and MNE, with respect to eLORETA. [file Image_2.TIFF]

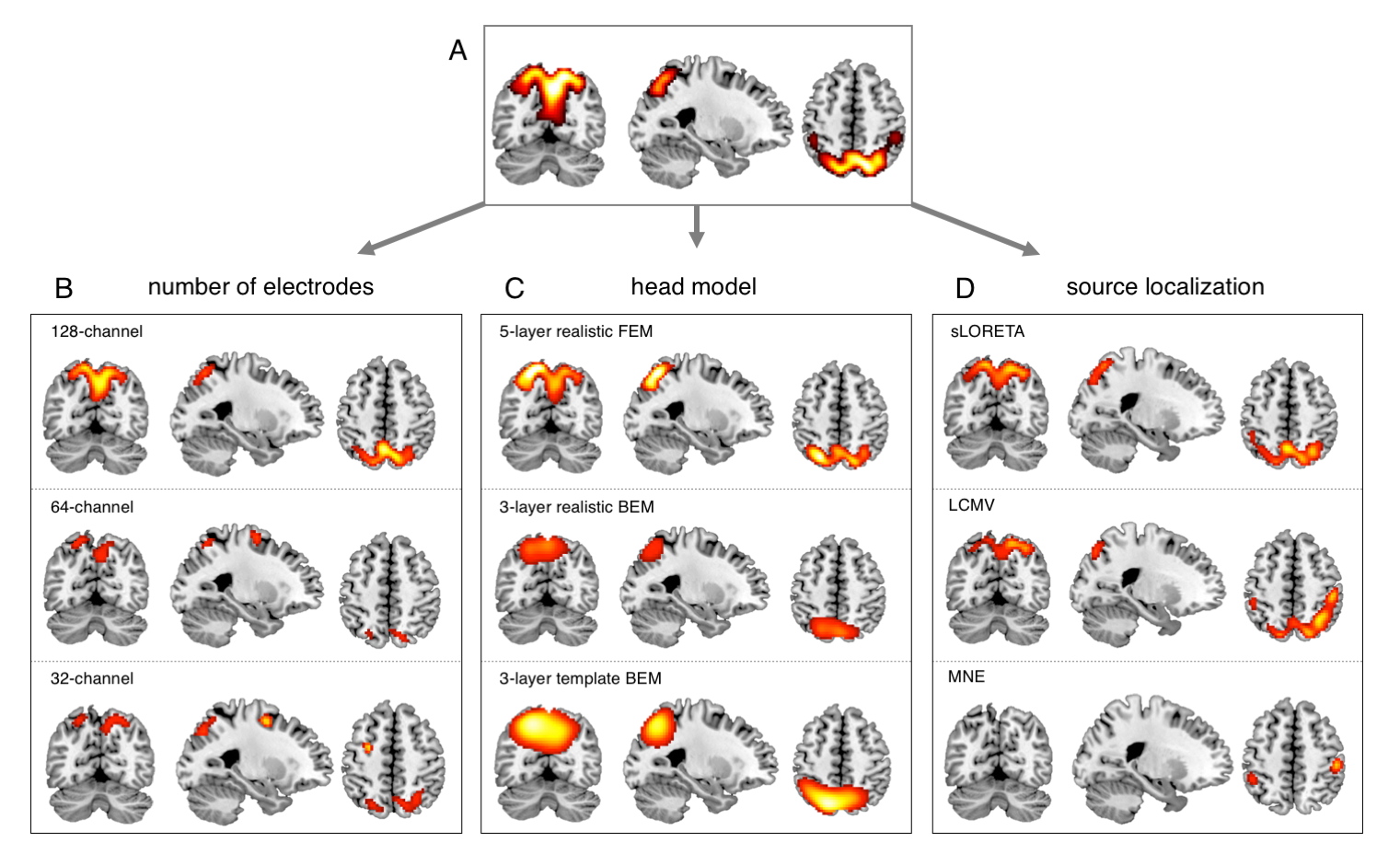

Supplement: FIGURE S3 — Influence of EEG montage density, head modeling and source localization on DAN reconstruction. EEG-RSNs were obtained: (A) using 256-channel recordings, 12-layer realistic FEM and eLORETA source localization. (B) Using 128-, 64-, and 32-channel recordings, respectively, with 12-layer realistic FEM and eLORETA source localization. (C) Using 5-layer realistic FEM, 3-layer realistic BEM and 3-layer template BEM, respectively, with 256-channel recordings and eLORETA source localization. (D) Using sLORETA, LCMV and MNE localization, respectively, with 256-channel recordings and 12-layer realistic FEM. [file Image_3.TIFF]

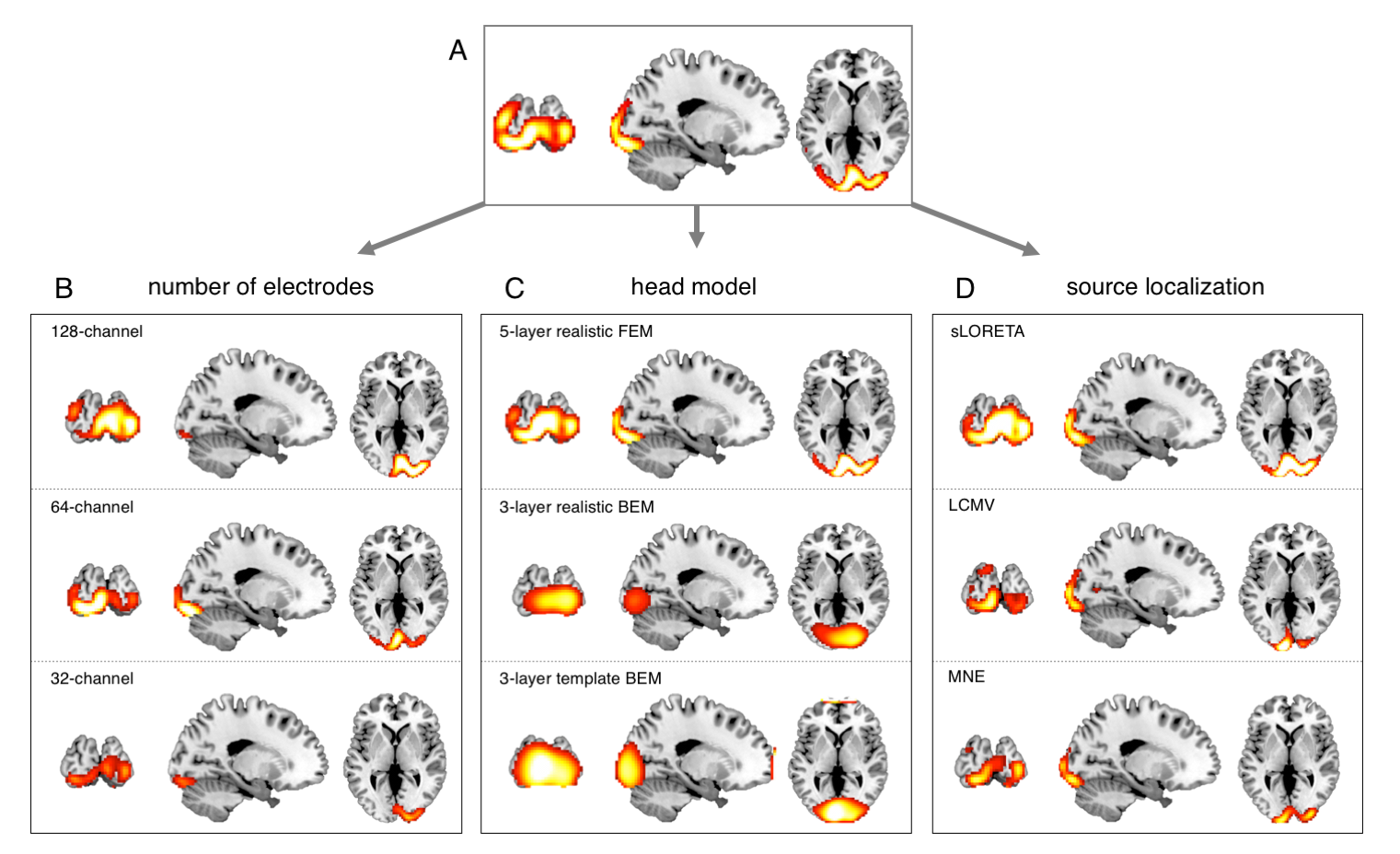

Supplement: FIGURE S4 — Influence of EEG montage density, head modeling and source localization on VFN reconstruction. EEG-RSNs were obtained: (A) using 256-channel recordings, 12-layer realistic FEM and eLORETA source localization. (B) Using 128-, 64- and 32-channel recordings, respectively, with 12-layer realistic FEM and eLORETA source localization. (C) Using 5-layer realistic FEM, 3-layer realistic BEM and 3-layer template BEM, respectively, with 256-channel recordings and eLORETA source localization. (D) Using sLORETA, LCMV, and MNE localization, respectively, with 256-channel recordings and 12-layer realistic FEM. [file Image_4.TIFF]

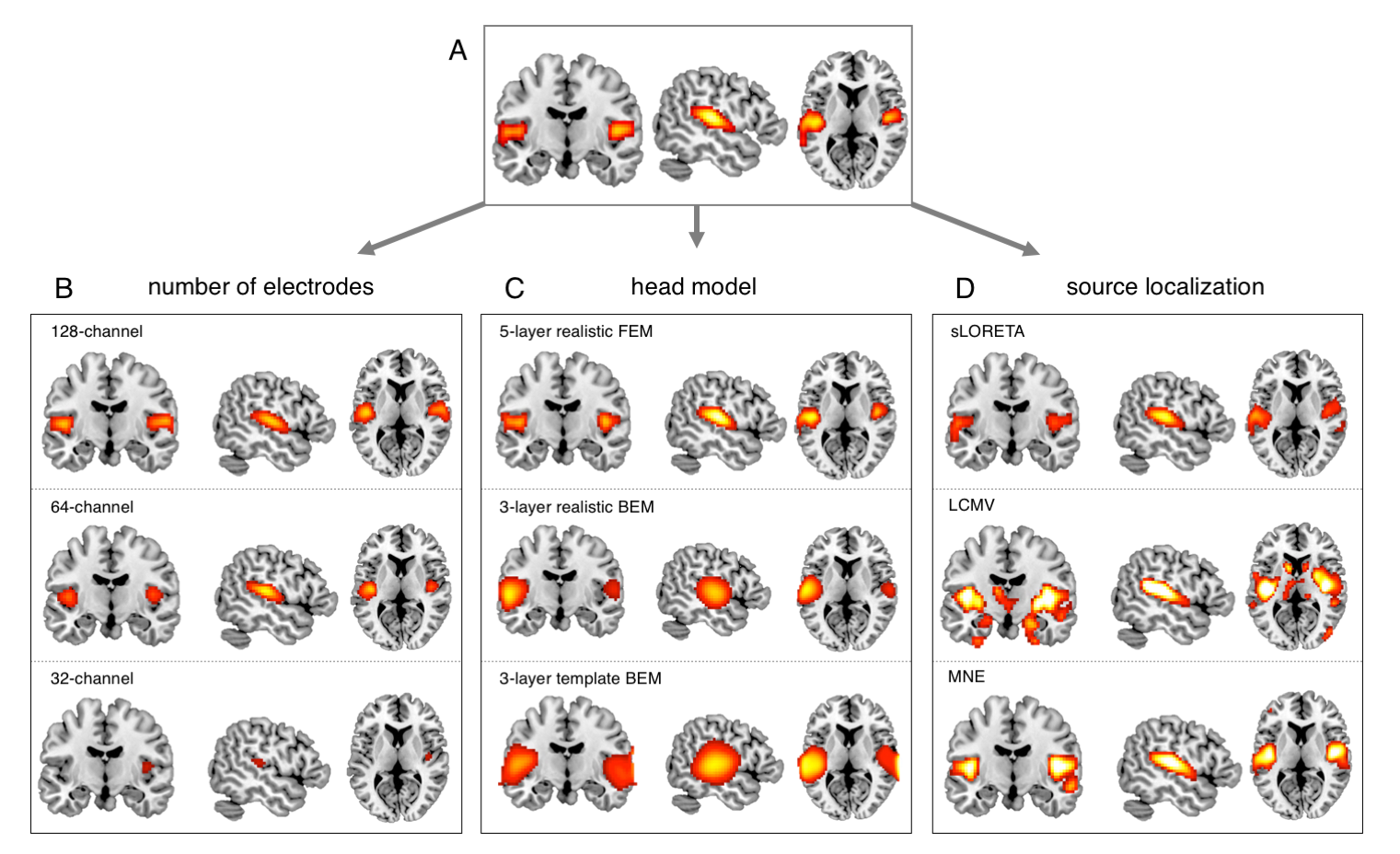

Supplement: FIGURE S5 — Influence of EEG montage density, head modeling and source localization on AN reconstruction. EEG-RSNs were obtained: (A) using 256-channel recordings, 12-layer realistic FEM and eLORETA source localization. (B) Using 128-, 64-, and 32-channel recordings, respectively, with 12-layer realistic FEM and eLORETA source localization. (C) Using 5-layer realistic FEM, 3-layer realistic BEM and 3-layer template BEM, respectively, with 256-channel recordings and eLORETA source localization. (D) Using sLORETA, LCMV, and MNE localization, respectively, with 256-channel recordings and 12-layer realistic FEM. [file Image_5.TIFF]

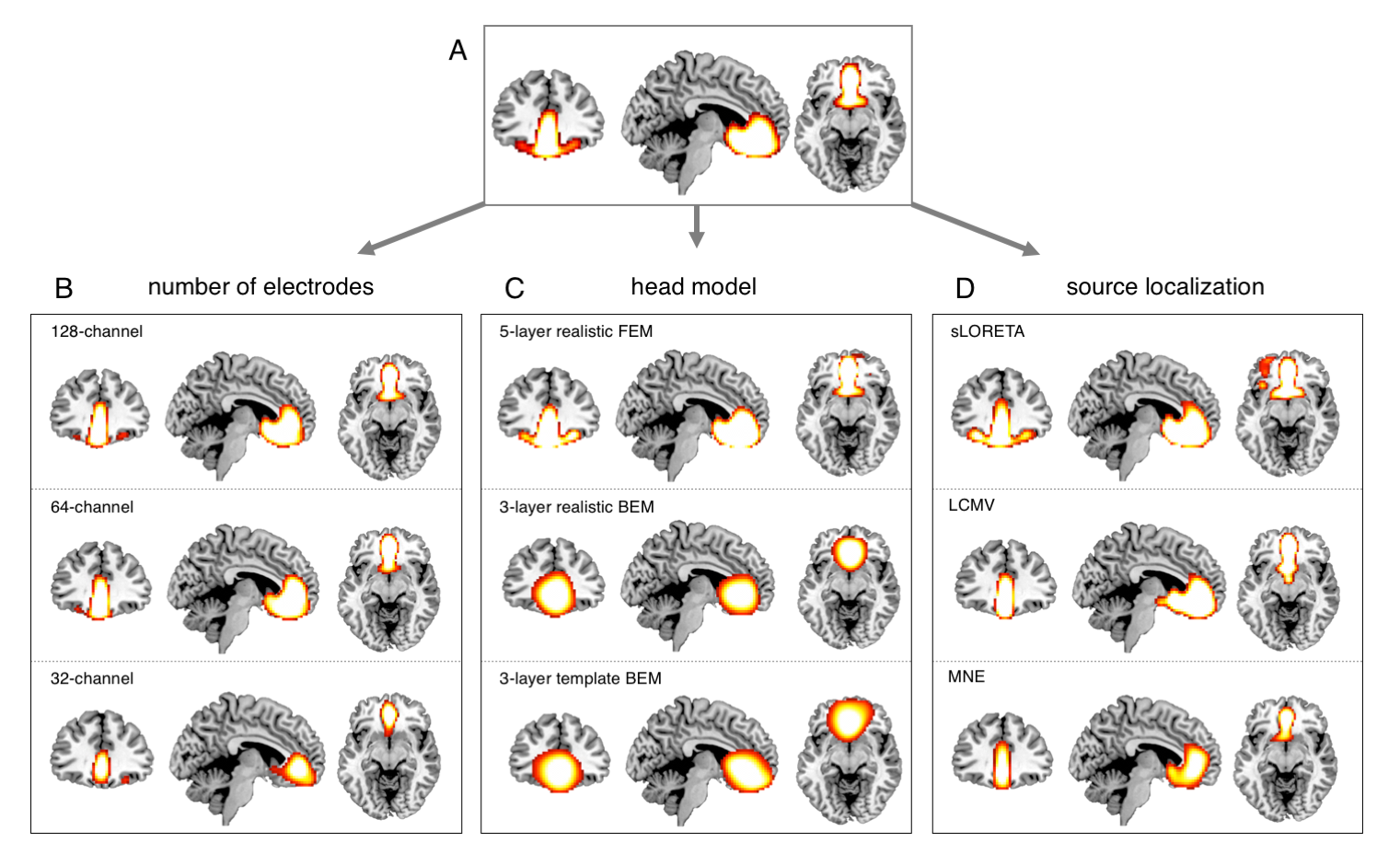

Supplement: FIGURE S6 — Influence of EEG montage density, head modeling and source localization on MPN reconstruction. EEG-RSNs were obtained: (A) using 256-channel recordings, 12-layer realistic FEM and eLORETA source localization. (B) using 128-, 64-, and 32-channel recordings, respectively, with 12-layer realistic FEM and eLORETA source localization. (C) Using 5-layer realistic FEM, 3-layer realistic BEM and 3-layer template BEM, respectively, with 256-channel recordings and eLORETA source localization. (D) Using sLORETA, LCMV and MNE localization, respectively, with 256-channel recordings and 12-layer realistic FEM. [file Image_6.TIFF]
