# Supplementary material for: Detecting Large-Scale Brain Networks Using EEG: Impact of Electrode Density, Head Modeling and Source Localization
Source: Front Neuroinform. 2018 Mar 2;12:4. doi: 10.3389/fninf.2018.00004 (PMC5841019; doi:10.3389/fninf.2018.00004)
Supplement: TABLE S1 — Conductivity values of different tissues used for the calculation of the head model. The conductivity values associated with the tissue classes were extracted from relevant literature (Haueisen et al., 1997). [file Table_1.PDF]

Supplementary Table 1. Conductivity values of different tissues used for the calculation of the head model. The conductivity values associated with the tissue classes were extracted from relevant literature (Haueisen et al., 1997).

Tissue name	Conductivity (S/m)
Skin	0.4348
compact bone	0.0063
spongy bone	0.0400
CSF	1.5385
cortical gray matter	0.3333
cerebellar gray matter	0.2564
cortical white matter	0.1429
cerebellar white matter	0.1099
brainstem	0.1538
eyes	0.5000
muscle	0.1000
fat	0.0400
